# Paediatric robotic surgery: a narrative review

**DOI:** 10.1007/s11701-023-01523-z

**Published:** 2023-01-16

**Authors:** Lukas Padraig O’Brien, Enda Hannan, Brice Antao, Colin Peirce

**Affiliations:** 1 Department of Paediatric Surgery, Children’s Health Ireland at Crumlin, Dublin, Ireland; 2 Department of Colorectal Surgery, University Hospital Limerick, St Nessan’s Road, Dooradoyle, Limerick, Co Limerick Ireland; 3 School of Medicine, University of Limerick, Limerick, Ireland

**Keywords:** Paediatric robotic surgery, Paediatric surgery, Robotic surgery, Minimally invasive surgery

## Abstract

The benefits of minimally invasive surgery (MIS) compared with traditional open surgery, including reduced postoperative pain and a reduced length of stay, are well recognised. A significant barrier for MIS in paediatric populations has been the technical challenge posed by laparoscopic surgery in small working spaces, where rigid instruments and restrictive working angles act as barriers to safe dissection. Thus, open surgery remains commonplace in paediatrics, particularly for complex major surgery and for surgical oncology. Robotic surgical platforms have been designed to overcome the limitations of laparoscopic surgery by offering a stable 3-dimensional view, improved ergonomics and greater range of motion. Such advantages may be particularly beneficial in paediatric surgery by empowering the surgeon to perform MIS in the smaller working spaces found in children, particularly in cases that may demand intracorporeal suturing and anastomosis. However, some reservations have been raised regarding the utilisation of robotic platforms in children, including elevated cost, an increased operative time and a lack of dedicated paediatric equipment. This article aims to review the current role of robotics within the field of paediatric surgery.

## Introduction

The advent of minimally invasive surgery (MIS) represents one of the most important surgical developments of the modern era and has seen significant growth and development over the past 30 years [1]. The benefits of MIS compared with traditional open surgery are well recognised [1]. These include a reduction in post-operative pain, inpatient length of stay, wound complications, improved cosmesis and an earlier return to normal activity [2]. MIS techniques were quickly embraced by adult general surgeons following the first adult laparoscopic cholecystectomy in 1987 by Philippe Mouret [3]. This in turn led to rapid advancements in complexity of surgery performed by MIS as expertise and skillset evolved with increasing volume and comfort, with complex major surgery by MIS now being the gold standard in adult patients [3, 4]. In contrast to this, utilisation of MIS in the paediatric community has progressed at a much slower rate [5, 6]. A significant barrier for paediatric MIS has been the technical challenge posed by laparoscopic surgery in small working spaces, where clashing of instruments and restrictive working angles may act as a barrier to safe dissection [5–7]. Thus, open surgery remains relatively commonplace in paediatrics, with significant debate existing over utilisation of laparoscopy even in index operations such as appendicectomy or inguinal hernia repair [5–7]. Significant controversy also exists regarding whether or not a high-fidelity oncologic resection of childhood malignancy can be achieved via laparoscopic surgery [6].

The limitations of laparoscopic surgery are well described [8, 9]. These include an unstable two-dimensional view, exaggerated tremor, limited ergonomics and reduced dexterity offered by rigid instruments [8, 9]. Such limitations become even more pronounced in smaller working spaces, and thus may be more apparent in smaller paediatric patients [5–7]. Robotic surgical platforms were developed to overcome the limitations of laparoscopic surgery [10]. This is achieved by offering a stable three-dimensional view, improved ergonomics, tremor elimination and greater range of motion [10]. Such advantages may be particularly beneficial in paediatric surgery by empowering the surgeon in the limited working space of a small abdominal or thoracic cavity [11]. Despite this, robotic platforms are currently not widely used in paediatric surgery, with issues relating to cost, operative time, availability and the lack of dedicated paediatric equipment being frequently quoted as barriers to utilisation [11, 12]. The purpose of this article is to provide a comprehensive and up to date review of the current state of robotic surgery in paediatric patients.

## Urology

Arguably the most significant uptake of robotics within the realm of paediatric surgery has been witnessed in urology [13, 14, 99]. This follows a similar trend as seen in adult surgery [10]. One of the first described robotic operations performed in children was a pyeloplasty for pelviureteric junction obstruction performed by Peters et al. in 2002 [13, 15, 16]. In this case, the author specifically noted that the robot platform was favourable due to the significant technical challenge in creating a ureteropelvic anastomosis by means of conventional non-articulating laparoscopic instruments [15]. Following this, a wide range of urological procedures have been performed using robotic platforms in the paediatric population, including ureteral reimplantation, ureteroureterostomy, appendicovesicostomy, nephrectomy and nephroureterectomy [17]. In 2018, a bibliometric analysis by Cundy et al. categorised 151 publications reporting on 3688 paediatric robotic urological procedures performed in 3372 patients from 2003 to 2016 [17]. This analysis revealed that the most common application was pyeloplasty (*n* = 1923) followed by ureteral reimplantation (*n* = 1120), with these two procedures dominating the literature (83%) [17].

### Robotic-assisted pyeloplasty (RAP)

The first paediatric laparoscopic pyeloplasty was performed in 1995, at which point the technique was noted to be highly technically challenging with a very steep learning curve due to the challenge of intracorporeal suturing in a restricted working space [18, 19]. Following the first paediatric RAP by Peters et al. in 2002, the inherent benefits of the robotic platform for this procedure became apparent, with a three-dimensional view and articulating instruments anecdotally allowing for greater precision in suturing and anastomosis formation [15, 20]. Numerous authors have subsequently reported a shorter learning curve for RAP compared with a laparoscopic approach [14, 21]. In most studies, success rates of greater than 90% have been widely reported with the technique [19]. In 2014, a meta-analysis of 12 retrospective studies that compared RAP with open and laparoscopic techniques was published [22]. This demonstrated a higher rate of success in RAP compared to laparoscopic pyeloplasty and equivalence with open surgery. No difference in complication rates or re-operation was observed between the three modalities. As is frequently observed in robotic literature, RAP was associated with greater cost and a longer operative time. However, a statistically significant reduction in inpatient length of stay was demonstrated in RAP [22]. In 2016, a multicentre study comprising of 575 patients demonstrated a shorter hospitalisation period and reduced post-operative complication rate in RAP compared to laparoscopic pyeloplasty [23]. A further multicentre experience with 2219 patients also supported that RAP resulted in a statistically significant reduction in length of stay compared to open and laparoscopic surgery with otherwise equivalent post-operative outcomes [24]. Further studies have consistently reported that RAP have a shorter hospital stay but longer operative times [15, 21]. RAP has also proven successful in small infants, with two studies examining its application in patients under 10 kg showing success and complication rates equivalent with open surgery [18, 25].

### Ureteral reimplantation (UR)

UR is performed for the treatment of vesicoureteral reflux (VUR) [13]. While the standard operative approach has been by open surgery, UR now represents the second most commonly performed paediatric robotic procedure, with 81% of minimally invasive implantation procedures performed utilising the robotic platform [13, 26, 27]. Several published studies describe this technique as safe and effective. Kasturi et al. demonstrated resolution of VUR in 99.3% of patients, while a case-matched study by demonstrated equivalent outcomes with open surgery (97% vs 100%) [28, 29]. One multicentre study comprising of 260 patients across 9 institutions reported a VUR resolution rate of 87.9% and an overall complication rate of 9.6%, equivalent with open outcomes [30]. A further prospective study demonstrated a 93.8% rate of radiographic resolution of VUR [31]. Marchini et al. also reported no significant difference in post-operative outcomes when compared with open surgery [32]. A reduced length of stay and post-operative pain is also widely reported [29, 33].

### Nephrectomy

In paediatric urology, partial or complete nephrectomy is most commonly indicated for benign disease rather than malignancy and is most commonly performed by open approach [34]. Of those performed by MIS, conventional laparoscopic approach remains more common than robotic surgery [34]. In 2019, a two-centre study comparing open, laparoscopic and robotic approaches demonstrated comparable post-operative complication rates between all groups [35]. Unsurprisingly, an open approach was associated with greater post-operative pain, while both laparoscopic and robotic surgery had significantly longer operative times [35]. When directly comparing robotic and laparoscopic approaches, Malik et al. demonstrated equivalent lengths of stay and incidence of complications [36]. Two subsequent series of robotic nephrectomies reported the incidence of complications at 8.3% and 9.5% respectively, comparable with published laparoscopic and open outcomes [37, 38].

### Miscellaneous

Paediatric ureteroureterostomy is performed for a number of indications, including obstructed ureterocoele or duplex systems with an upper pole ectopic ureter [13]. A small number of case series report on successful robotic-assisted ureteroureterostomy [39–42]. One study, which made comparison with an open cohort, concluded that operative times and complication rates were comparable with a shorter length of stay for robotic cases [42]. Reconstructive bladder surgery, such as the ‘Mitrofanoff’ appendicovesicostomy, has also been demonstrated to be safe and feasible when performed by a robotic approach, with Grimsby et al. showing no difference in complication rates [43]. Successful cases of robotic excision of bladder diverticulum, prostatic utricles, varicocoele, seminal vesicle cyst, posterior urethral diverticulae and urachal cyst have all also been described in case reports and small case series [13, 44–46].

Currently, the robotic paediatric urology approach appears to offer similar outcomes and complication rates to open and laparoscopic approaches [13, 14, 99]. When compared directly with open surgery, robotic approaches appear to offer shorter lengths of stay and reduced postoperative pain [14]. Robotic paediatric urology does appear to come with greater cost and operative time, but is advantageous in procedures that require intracorporeal suturing, such as pyeloplasty [13–15]. As with adult urology, procedures that require access to the pelvis and thus have a narrow operative field may be particularly suited to the robotic approach [13].

## General surgery

Robotics have not yet reached the level of utilisation in paediatric general surgery that has been observed in paediatric urology [13, 14, 47]. Nonetheless, it is the field within which there has been the second greatest uptake of robotic technology in paediatric surgery [13, 14, 47]. The most common applications of the robot in paediatric general surgery have been in gastric fundoplication and choledochal cyst excision [13, 14, 47]. As both of these procedures demand precise intracorporeal suturing, robotic platforms may render this less challenging than utilising rigid non-articulating laparoscopic instruments in a restricted working space [47]. Other robotic procedures that have been described in the literature include hepatectomy, colectomy, proctectomy with ileal pouch-anal anastomosis, resection of mediastinal masses and congenital diaphragmatic hernia repair [14, 47].

### Gastric fundoplication

Fundoplication is the most commonly performed and reported robotic procedure in paediatric general surgery [14, 48]. In 2014, Cundy et al. published a meta-analysis comparing outcomes in robotic versus conventional laparoscopic fundoplication in children. Here, it was observed that laparoscopic procedures had a greater tendency towards conversion to open surgery than robotic surgery (6.1% vs 3%) while the incidence of post-operative complications was equivalent between the two cohorts; however, all included studies were limited by a lack of long-term follow-up [49]. A prior systematic review that compared 89 robotic fundoplications with 85 laparoscopic procedures showed a statistically significant reduction in post-operative complications in robotic cases, albeit with a longer operative time [50]. The authors theorised that the reduced complications may be a result of greater dexterity and precision within the subphrenic space [47, 50]. It has also been suggested that robotic surgery may be advantageous in challenging cases, such as those in obese patients, large hiatal defects and in cases of redo fundoplication, which are all recognised as being highly technically demanding with conventional laparoscopic approaches [47, 51, 52].

### Choledochal cyst excision

Minimally invasive hepatobiliary surgery in children, such as choledochal cyst resection with Roux-en-Y hepaticojejunostomy, is highly challenging and requires high levels of precision [53]. For this reason, it is unsurprising that many still elect to perform such procedures by open techniques [53]. In described laparoscopic techniques, anastomosis is often performed in an extracorporeal manner by extension of the umbilical incision, which may have a detrimental impact on the recovery benefits offered by MIS [53]. The ergonomic advantages and stability offered by a robotic platform may facilitate intracorporeal anastomosis in a manner that is not feasible by laparoscopic surgery and thus, limit the need for bowel exteriorisation [54]. This is an opinion which has been expressed by many with experience in both laparoscopic and robotic approaches [54–57]. Kim et al. retrospectively compared open and robotic techniques, concluding that there was no difference in the incidence of post-operative complications [58]. A shorter length of stay was noted in the robotic cohort, albeit with a statistically significant increase in operative time [58]. It is also important to note that those in the robotic group were significantly larger in size and of an older age, perhaps suggesting a larger workspace more favourable for MIS [58]. However, Dawrant et al. did demonstrate that a robotic approach was feasible in smaller children in a series of patients under 10 kg [59]. In 2018, Wang et al. published a review article that analysed a combined 86 cases from 8 studies, demonstrating a postoperative complication rate of 11.6% and conversion rate of 8.1% [60]. While this study lacked a control group, these outcomes would appear similar to those reported in open and laparoscopic modalities, with the added advantage of facilitating intracorporeal reconstruction [53].

### Surgical oncology

While robotics is widely used in adult oncological surgery, open techniques currently remain the standard of care for resection of paediatric abdominal tumours, with a lack of high-level evidence supporting the relatively recent development of robotic approaches [13, 47, 47]. Despite this, there does exist a wide range of literature mostly in the form of individual case reports or small case series. One case of successful robotic resection of a stage IV neuoroblastoma has been reported, with the authors noting that the enhanced vision and precision of the robotic platform allowed for skeletalisation of tumour vasculature that may not have been feasible laparoscopically [61]. Another case described the management of a 4 cm juvenile cystic adenomyoma by a robotic approach in a 15-year-old girl, with improved ergonomics allowing for four-layered closure of the uterus, followed by an uneventful post-operative recovery [62]. Anderberg et al. also reported on a robotic radical cystoprostatectomy for management of rhabdomyosarcoma in a 22-month old child weighing 8 kg, with the robot proving advantageous in the confines of the paediatric bony pelvis [63]. Successful robotic partial adrenalectomy for phaeochromocytoma in a child has also been described [64].

A common theme discussed in many of these cases is the advantages offered by a robotic approach to extended lymph node dissection resulting from enhanced 3-dimensional vision [13, 61–64]. A recent case series of 12 robotic resections of paediatric abdominal tumours concluded that oncological surgical principles were maintained by this approach, with all achieving R0 resection status, low post-operative morbidity and good long-term results. The authors concluded that robotic surgery brings potential benefits to children with cancer but its place and indications still need to be better defined [65]. Concerns regarding the adherence to sound oncological principles, with clear resection margins and avoiding tumour spillage, have been raised in relation to paediatric robotic surgery, with some theorising that the loss of haptic feedback affecting the surgeon’s ability to differentiate between tumour and normal tissue [13, 47]. However, it has equally been suggested that improved vision may compensate for this loss in tactile feedback [63]. Ultimately, long-term data are required to demonstrate whether oncological outcomes in paediatric robotic surgery are acceptable and this data is not currently available [47]. However, a well-recognised contraindication for laparoscopy in paediatric malignancy is large or fragile tumours that pose high risk of tumour spillage or fracture, and this should be respected in regard to robotic approaches also [47].

### Miscellaneous

Robotic cholecystectomy has been well described in paediatric literature, including both single-port and multi-port approaches, with the consensus that it is safe and effective, albeit costly and time-consuming [66–68]. Given that this offers no true benefit to a laparoscopic approach, it is difficult to advocate for routine robotic cholecystectomy [66–68]. Nonetheless, robotic cholecystectomy serves a valuable role as an introductory procedure for paediatric surgeons that wish to develop a robotic skillset and is widely supported as a training operation [66–68]. Similarly, while robotic splenectomy has been shown to be safe and effective, it offers no demonstrable benefit to the quicker and cheaper laparoscopic approach [69]. Conversely, it has been demonstrated that a robotic approach to Heller’s myotomy in children may be advantageous to laparoscopic surgery by a lower risk of inadvertent mucosal perforation [70, 71].

Similarly, it has been suggested that the robot may be advantageous in gynaecological surgery, with improved vision and ergonomics in the narrow bony pelvis in cases of paediatric ovarian tumours [72, 73]. The precision offered by robotics has also been suggested to be beneficial in maintaining ovarian morphology where possible, especially in benign disease, thus allowing for recovery in post-operative ovarian function [47, 73]. The advantages offered by the robot in pelvic dissection have also been reported in cases of robotic anorectal pull-through for anorectal disorders [74]. Robotics have also been demonstrated to be beneficial in the management of Hirschsprung’s disease, with a recent prospective series of robotic Soave pull-through procedures in patients under 12 months demonstrating low morbidity, a short inpatient length of stay and acceptable long-term outcomes [75].

Another example in the literature where robotic platforms have allowed paediatric surgeons to overcome limitations of laparoscopy is in the management of superior mesenteric artery syndrome by means of Roux-en-Y duodenojejunostomy [76]. In this case, the authors note that a robotic approach facilitated safe intracorporeal anastomosis in a manner that would be highly challenging laparoscopically [76].

## Cardiothoracic surgery

In the context of thoracoscopic surgery in children, which has continued to evolve over the past 3 decades, paediatric robotic-assisted thoracic surgery (RATS) is in its relative infancy, with significantly less published literature than in both urology and general surgery [13, 14, 47]. Nonetheless, early reports have been promising, with a reduction in learning curve noted in RATS compared with thoracoscopic surgery [47]. The recovery benefits of minimally invasive thoracic surgery are well documented, and it has been reported that MIS also reduces risk of spinal and thoracic deformity in children following lung resection [47].

### Thoracic surgery

Lobectomy is the most widely reported RATS in paediatric patients. First described in 2006, multiple case series with modest patient cohorts have since shown equivalent post-operative outcomes with thoracoscopic surgery and a quicker postoperative recovery than open surgery, albeit with a prolonged operative time than both approaches [77–79]. Successful cases of robotic congenital diaphragmatic hernia repair, both via thoracic and abdominal approaches, have been reported, with the authors stating preference for a robotic approach over thoracoscopic and laparoscopic techniques, which render satisfactory closure of the diaphragmatic defect challenging [80]. Other successfully described RATS procedures include thymectomy for treatment of myasthenia gravis, resection of bronchogenic cysts and tracheopexy for tracheomalacia [81–83]. A consistent theme in RATS literature, however, appears to be equivalent outcomes to thoracoscopic surgery albeit with a longer operative time, although many authors anecdotally note improved ergonomics and a shallower learning curve [47, 77–83]. In one series of 11 patients, it was noted that the neonatal thorax represented an obstacle in adapting 5 mm or 8 mm ports required for most robotic platforms, with the conclusion that RATS should be reserved for patients weighing more than 20 kg [84]. With regard to the management of thoracic tumours, it has been noted that the robot may be well adapted to the required intricate mediastinal dissection for a safe minimally invasive approach, with the authors of one series noting that RATS allowed for better visualisation of the tumour and its anatomic connections than typically experienced even in open surgery [85].

### Cardiac surgery

Currently, experience with robotic platforms in the management of cardiac conditions is limited. In one study, which examined RATS for the division of congenital vascular rings, the conclusions was that while both safe and effective, RATS offered no demonstrable benefit to video-assisted thoracic surgery (VATS) [86]. Similarly, in a retrospective study of paediatric patients with patent ductus arteriosus, RATS was noted to take longer than VATS without any difference in post-operative outcomes [87]. Hassan et al. described a case of robotic excision of a left ventricular myxoma in a child, concluding that the technique is safe and feasible [88].

## Ear, nose and throat surgery

The most frequent application of robotics in otorhinolaryngology has been in transoral approaches which have proved beneficial in accessing base of tongue lesions in a manner that limits morbidity and improves cosmetic outcomes [47, 89]. Typically, access to the oropharynx would require pharyngotomy or division of the lip and jaw [89]. The robotic transoral approach avoids the potential disfigurement and pain associated with such access [89]. A case series consisting of 41 paediatric patients managed by a robotic transoral approach for a variety of indications, including oropharyngeal sarcoma and laryngeal cleft cysts, showed encouraging results, with more than 90% of cases completed successfully without conversion and low post-operative morbidity [90]. While still a relatively novel approach, it has been suggested that robotic transoral surgery may become the standard of care for base of tongue lesions [90].

## Neurosurgery

The utilisation of robotic technology in neurosurgery has been described in the form of the robotised stereotactic assistant, or ROSA®, whereby a computer-controlled robotic arm with an integrated platform that combines image-guided neurosurgical planning software with robotic navigation to assist neurosurgeons with minimally invasive procedures, such as deep brain stimulation lead placement, stereotactic biopsies, laser ablation of epileptogenic foci, endoscopic third ventriculostomy and electrode placement for seizure monitoring [91]. ROSA® has generated particular interest in the paediatric population. As a child’s developing brain is extremely vulnerable to injury, an accurate image-guided minimally invasive approach to paediatric neurosurgery is highly desirable [91]. The largest published case series, consisting of 123 children managed with ROSA® for a variety of indications, showed a high rate of success (97.7%) with low post-operative morbidity (3.9%). No patients in this series experienced any long-term neurological deficit [92].

The robot has seen similar applications in paediatric spinal surgery, where it plays a role in accurate placement of surgical prostheses supported by image-guided software [93]. A recent development has been the management of idiopathic scoliosis in children by robotic-assisted placement of pedicle screws [93]. It has been demonstrated that this utilisation of robotic technology can reduce the incidence of pedicle malposition, a complication seen more commonly in paediatric populations owing to a smaller size of pedicle and target location than in adults [93, 94]. Incidence of pedicle screw malposition has been reported to be as high as 17.9% previously, but with image-guided robotic assistance, an accuracy of 97.6% in screw placement has been demonstrated in a recent literature review [93].

## Benefits and limitations

### Benefits

All of the benefits that laparoscopic surgery offer in comparison to open surgery also apply to robotics, with reduced post-operative pain, reduced opioid requirements, improved cosmesis, a shorter inpatient length of stay, reduced wound complications and a faster return to normal activities [2]. However, advocates of robotic surgery argue that the inherent characteristics of the robotic platform allow it to supersede the minimally invasive capabilities of traditional laparoscopic surgery [47]. Robotic instruments have been specifically designed to emulate the range of movements possible with a human wrist, as opposed to the restricted movements available with standard long, rigid laparoscopic instruments that are incapable of bending [10, 11]. This enhanced dexterity may be particularly advantageous in the reduced working space of smaller paediatric patients, making steps such as intracorporeal suturing or anastomosis possible in a way that may either be technically impossible or highly challenging with laparoscopic instruments [5–7]. Further to this, robotic platforms are equipped with motion scaling, which acts to reduce the scale of the surgeon’s movements 5:1, allowing for greater precision in smaller cavities [47]. It has also been suggested that robotic surgery may offer a gentler learning curve than traditional laparoscopic surgery [94, 95]. This has been attributed to the symmetrical movements of robotic instruments with the surgeon’s hands, unlike laparoscopy that requires inverted movements [47]. Rapidly decreasing operative times in robotic surgery with experience have been widely observed [94, 95].

Clear visualisation of paediatric anatomy can prove highly challenging with traditional laparoscopic cameras, where an unstable two-dimensional view not controlled by the primary surgeon may create a barrier to clear identification of critical structures and planes [8, 9]. Even in traditional open surgery, paediatric surgeons may struggle with visualisation, with the use of surgical loupes often required [47]. Robotic platforms are capable of magnifying images between 10 and 15 times, which is further enhanced by 3-dimensional vision, tremor elimination and operator-controlled views [47]. This yields steadier and more precise visualisation with enhanced depth perception [47].

### Limitations

Frequent points of criticism aimed at robotic surgery have been in relation to both an increased cost of surgery as well as a longer operating time compared to traditional laparoscopic surgery, and these points are equally applicable in the realm of paediatric robotic surgery [11, 12]. A variety of factors contribute to a longer operative time in robotic surgery, including time spent with setup of the robotic platform and for troubleshooting; it has been shown that this shortens significantly with time and experience [95]. A disadvantage of robotic surgery specific to paediatrics relates to the size of the surgical robotic platforms and associated instruments [47]. Robotic instruments approved for paediatric use are usually only available in two sizes (8 mm and 5 mm), both of which are larger than 3 mm instruments typically used in laparoscopic procedures for smaller paediatric patients [47]. Similarly, robotic cameras typically exist in 12 mm and 8 mm sizes, with a previously utilised 5 mm endoscope having been discontinued due to low utilisation [96]. While the 8 mm endoscope may be appropriate in many paediatric patients, it is possible that this is prohibitively large in some children, particularly in cardiothoracic surgery where the port must fit between the confines of the intercostal space [47, 97]. It is also recommended for the da Vinci platform that ports be placed 6–10 cm apart, which may be difficult to achieve in small children [96].

The Senhance platform (Transenterix) does have 3 mm instruments available, and while this has not yet been approved for use in paediatric patients, laboratory based experimentation utilising these instruments within boxes designed to mimic the dimensions of paediatric abdomens have shown that high precision tasks, such as intracorporeal suturing and knot-tying, have been achievable in cavities with a volume as small as 90 ml [98]. This platform also allows for direct insertion of these 3 mm instruments into the abdomen without ports, reducing the necessary distance between insertion points [14]. The Senhance platform also offers haptic feedback [98].

## Conclusion

This review demonstrates the use of robotic platforms for paediatric surgery as an exciting and promising development that may allow children to benefit from the advantages of MIS, particularly in cases where the limitations of rigid laparoscopic instruments are prohibitively restrictive in the smaller working spaces found in children. Particular interest in robotic techniques has been observed in paediatric urology and general surgery, where the ergonomic advantages prove advantageous in procedures that require intracorporeal suturing and anastomosis. It is evident from this review that paediatric robotic surgery is currently still in its infancy, with larger and more robust prospective studies needed to truly ascertain the benefits and limitations of this approach in comparison to open and laparoscopic surgery. Nonetheless, paediatric robotic surgery offers great potential to allow a young and very vulnerable patient cohort to benefit from the advantages of MIS supported by the improved ergonomics and dexterity afforded by robotics in reduced working spaces.

## Abbreviations

MIS: Minimally invasive surgery
RAP: Robot-assisted pyeloplasty
UR: Ureteral reimplantation
RATS: Robot-assisted thoracoscopic surgery
VATS: Video-assisted thoracoscopic surgery
